# A Case of Acute Decompensated Heart Failure Complicated by Cardiorenal Syndrome Treated With Ultrafiltration

**DOI:** 10.7759/cureus.29013

**Published:** 2022-09-10

**Authors:** Gaurav Mandal, Tarig Elraiyah, Charn Nandra, Rick Greco, John Schirger

**Affiliations:** 1 Internal Medicine, Trinity West Medical Center, Steubenville, USA; 2 Cardiology, Trinity West Medical Center, Steubenville, USA; 3 Cardiology/Advanced Heart Failure and Transplant, Trinity West Medical Center, Steubenville, USA

**Keywords:** management of heart failure, ultrafiltration, diuresis, cardiorenal syndrome type i, decompensated heart failure

## Abstract

The standard of care of treatment for acute decompensated heart failure (ADHF) complicated by cardiorenal syndrome (CRS) is diuretics. This case report is an example of how the institution of ultrafiltration early in the course of ADHF complicated by CRS for volume removal can be an alternative approach rather than escalation of the diuretic regimen when the initial diuretic regimen is ineffective and, in turn, yielding a good clinical course.

## Introduction

The standard of care for treating volume overload in patients with acute decompensated heart failure (ADHF) is using loop diuretics with or without thiazide-type diuretics [1-2]. However, some randomized control trials, such as the Aquapheresis Versus Intravenous Diuretics and Hospitalization for Heart Failure (AVOID-HF) study, and the Ultrafiltration versus Intravenous Diuretics for Patients Hospitalized for Acute Decompensated Congestive Heart Failure (UNLOAD) study used ultrafiltration (UF) as an alternative to diuretics for volume removal during hospitalization with ADHF, and reported a decrease in the number of first heart failure events after hospital discharge in favor of UF [3,4]. ADHF can also be associated with cardiorenal syndrome (CRS), a spectrum of disorders involving the heart and kidneys in which dysfunction of one organ may result in dysfunction of the other [5]. According to the 2013 American College of Cardiology (ACC) and American Heart Association (AHA) guidelines, treating patients with ultrafiltration (UF) is a Class 2B or a weak recommendation in treating ADHF complicated by CRS, and UF can be only be employed as a strategy for volume removal when diuresis is not successful [6]. In this report, we present a challenging case of new-onset heart failure with hypervolemic signs and symptoms complicated by CRS that were resistant to medical treatment.

## Case presentation

**Figure 1:**
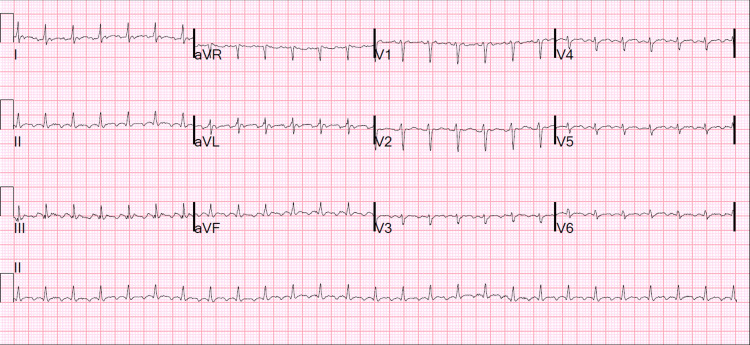
EKG showing 2:1 conduction atrial flutter with rapid ventricular response, low QRS voltage in precordial leads, and poor R-wave progression

**Figure 2:**
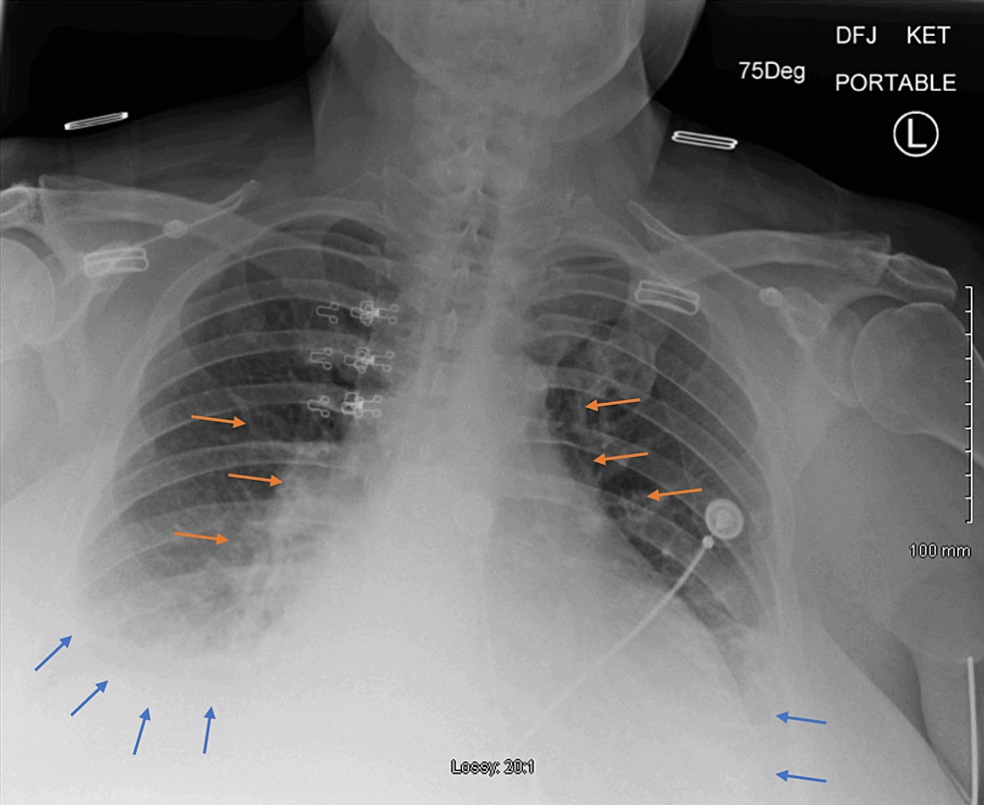
Chest x-ray showing mild cardiomegaly with interval development of bilateral pleural effusions (blue arrows) with bibasilar atelectasis, pulmonary vascular congestion, and edema (red arrows).

A 45-year-old Caucasian female presented to the hospital with complaints of worsening shortness of breath over the past two weeks associated with orthopnea, palpitations, massive lower extremity edema, and subjective weight gain of about 20 pounds in the preceding two months. Past medical history included morbid obesity, May-Thurner syndrome with stents to the right external iliac, right common femoral and left common femoral veins and chronic venous stasis ulcers. Physical exam was significant for morbid obesity, jugular venous distention, bilateral basilar crackles, bilateral lower extremity edema that extends to the torso, and irregular heart rhythm. Vital signs were significant for hypotension, tachycardia, and hypoxia. Her weight was 345 pounds, and her last measured weight was 276 pounds about three months earlier. Laboratory testing revealed elevated brain natriuretic peptide (BNP) of 866 pg/ml, normal troponin, and elevated creatinine (Cr) of 1.7 mg/dl from a baseline of 0.80 mg/dl. EKG showed atrial flutter with a rate of 157 bpm (Figure 1). Chest x-ray showed interval development of mild congestive heart failure (CHF) (Figure 2).

The patient was initially placed on a norepinephrine drip for hypotension and was given furosemide 20 mg intravenous (IV) once for volume overload status. She was admitted to the intensive care unit for management of acute hypoxic respiratory failure secondary to pulmonary edema in the setting of suspected new onset CHF, and atrial flutter with a rapid ventricular response.

On day two, the patient’s O2 requirement increased to 6 L nasal cannula. Central venous pressure (CVP) was noted to be 30 mmHg. Echocardiography showed biventricular heart failure with an estimated ejection fraction of 35%, pulmonary hypertension, and tricuspid and mitral regurgitations. The patient was in cardiogenic shock, and thus dopamine was added to the vasopressor regimen. The patient was initially treated with a regimen of diuretics that consisted of furosemide 40 mg every four hours. Beta blockade was started for rate control and heparin drip was started for anticoagulation for her atrial flutter/fibrillation.

On day three, the patient’s O2 requirement increased to 60 L/min delivered by a heated/humidified high-flow nasal cannula system. CVP was noted to be 35 mm Hg. Cr increased to 2.6, bicarbonate dropped to 15, potassium increased to 5.5 mEq/L, and lactic acid increased to 6.5 mg/dl. There was no improvement in urine output (UOP) and it was only 400 mL for the first three days combined. Fractional excretion of urea was 9.1% suggestive of prerenal azotemia, which was thought to be developing in the setting of cardiorenal syndrome (CRS). Instead of escalating the diuretic regimen, the decision was made to initiate continuous renal replacement therapy (CRRT) primarily for fluid removal. Phenylephrine was required for additional vasopressor support. The beta blocker was discontinued owing to persistent shock, and one dose of digoxin was given.

On days four to six, the patient responded satisfactorily to UF with net volume removal of approximately 4-6 L per day. She was able to be weaned off the high-flow oxygen gradually to room air. Serum Cr improved from 2.52 to 1.81, and finally to 1.71 over this period of time. Beta blockade was reinstituted and the dosages were uptitrated as tolerated.

On days 7-12, CRRT was switched to intermittent hemodialysis with UF as needed. The patient started to have progressively improving urine output from 500 mL to 1300 mL per day during this time. Her blood pressure improved, and vasopressors were discontinued on day nine. For her atrial fibrillation, cardioversion was considered but was put on hold due to the fact that she tested positive for coronavirus disease 2019 (COVID-19). As such, the patient was started on oral anticoagulation with apixaban owing to her CHADSVASC (congestive heart failure, hypertension, age, diabetes mellitus, prior stroke, vascular disease, age, sex category) score of 3.

On day 13, the UF was discontinued, and the patient was transitioned to a diuretic regimen composed of bumetanide 2 mg IV twice daily and metolazone 5 mg by mouth once daily. With this regimen, she had a very good UOP of about 1.5-4.0 L per day. In the remaining hospital days, her Cr improved to her baseline level. She experienced significant improvements in her renal, respiratory, and circulatory functions. Her weight on the day of discharge was approximately 300 pounds. No significant adverse events such as venous access site discomfort, UF filter clot, central venous catheter infection, or bleeding were observed in association with UF therapy [3]. Guideline-directed medical therapy for heart failure with reduced ejection fraction was added gradually including spironolactone, empagliflozin, and lisinopril. Amiodarone was added for rhythm control as well, and the patient was discharged with a plan to follow up outpatient for cardioversion.

## Discussion

UF is a Class 2B or a weak recommendation according to the ACC/AHA for volume removal in the setting of CRS based on the 2012 Cardiorenal Rescue Study in Acute Decompensated Heart Failure (CARRESS-HF) trial [7]. This trial demonstrated that pharmacologic treatment was superior in both efficacy and safety to UF therapy in treating patients with ADHF complicated by CRS. The mainstay therapy was escalating the dose of diuretics when initial dosages result in inadequate diuresis [7]. Nonetheless, the patient in this case report responded favorably to the UF strategy after failing a trial of loop diuretic therapy. Instead of increasing the dose of loop diuretics and adding thiazide-type diuretics, the management was escalated to UF for her volume overload status to relieve her congestive heart failure symptoms. Post UF, the patient responded very well to the institution of diuretics. She had a urine output of 1.5-4.0 L per day, suggesting the restoration of diuretic response in the setting of being diuretic resistant initially on days two and three despite escalating the furosemide dose 40 mg IV every four hours. Similar responses to diuretics were also observed recently post UF in a randomized control trial [8]. In our patient, it was evident that early utilization of UF had led to the resolution of the cardiogenic shock from a newly diagnosed HF, acute kidney injury, and acute respiratory failure. The significant improvement in CHF symptoms, exercise tolerance, blood pressure, and cardiac output can be attributed to the removal of isotonic fluid by UF rather than hypotonic fluid diuresis with loop diuretics [4,8].

Of note, we used a similar UF strategy to the one described in the AVOID-HF trial. This strategy dictates titrating the UF according to the patient's hemodynamics. This approach resulted in full recovery of the patient’s renal function as mentioned above. In contrast, the UF rate in the landmark CARRESS-HF study was fixed at 200 mL/hour, which could explain the worsening of renal function in patients with ADHF with CRS observed 96 hours into the study (mean change in Cr of 0.04±0.53 mg/dL decrease vs. 0.23 mg/dL increase) [7]. Notably, the ACC/AHA 2013 guidelines for ADHF management, concluded that UF is a Class 2B recommendation. In contrast, the updated 2022 guidelines did not comment on UF as one of the treatment options for ADHF complicated by CRS [2,7]. Despite the evidence supporting medical treatment for ADHF complicated by CRS, some patients may respond better to the UF strategy, as shown in this case report. Hence, this should be further explored in more extensive prospective studies on these patients, which may refine our management guidelines.

The use of UF rather than medical management can be appropriate in hospitalized patients with ADHF who have acute hypoxic respiratory failure and have confirmed diuretic resistance. The patient can be considered resistant to diuretics if they did not respond to a dose escalation regimen beyond a person's previously known ceiling dose, or a dose approaching the maximum recommended daily dose without incremental improvement in urine output. It should be noted that a higher percentage of patients in the UF group than in the pharmacologic-therapy group had serious adverse events over the 60 days of follow-up in the CARRESS-HF trial [7]. Hence, treatment has to be highly individualized when adopting this dual strategy.

This case highlights the importance of implementing an individualized treatment plan that considers the specific patient’s characteristics and clinical scenario. The case emphasizes the significance of adopting a personalized approach while utilizing the strategies of evidence-based medicine to inform our clinical decision-making. Embracing this dual approach will likely ultimately lead to high-quality patient-centered care and improve patients’ satisfaction with their outcomes.

## Conclusions

Diagnosis and treatment of patients with ADHF complicated by CRS are challenging and require a careful, individualized approach. Despite high-quality evidence supporting medical treatment for CRS, some patients may respond better to the UF strategy. As such, clinical providers should consider an alternative therapeutic approach for patients who have failed the standard of care management.
